# Influence of the COVID-19 pandemic on the amputation rate in Germany in patients with critical limb-threatening ischaemia

**DOI:** 10.1515/iss-2024-0035

**Published:** 2024-12-18

**Authors:** Wojciech Derwich, Oliver Schöffski, Eva Herrmann, Kyriakos Oikonomou

**Affiliations:** Vascular and Endovascular Surgery of Department of Cardiac and Vascular Surgery, University Hospital Frankfurt Goethe University, Frankfurt/Main, Germany; School of Business and Economics, Chair of HealthCare Management, Friedrich-Alexander-University of Erlangen-Nürnberg, Nürnberg, Germany; Institute for Biostatistics and Mathematical Modeling, Goethe University Frankfurt, Frankfurt/Main, Germany

**Keywords:** COVID-19, CLTI, limb amputation

## Abstract

**Objectives:**

The COVID-19 pandemic has shown a wide range of direct and indirect influences on the health of the population. This study examines the impact of the COVID-19 pandemic on the amputation rate in Germany in patients with critical limb-threatening ischaemia (CLTI).

**Methods:**

The epidemiological, pooled, and anonymised data of 476,168 CLTI patients from the Institute for the Hospital Remuneration System (InEK) were analysed for 2019–2023 at the federal level at weekly intervals and compared with epidemiological data for COVID-19 patients from the Robert Koch Institute (RKI) database, divided into the pandemic phases.

**Results:**

The number of inpatient cases declined by −4.64 % (p>0.05) from 2019 to 2020 and increased significantly by +7.07 % (p=0.001) from 2020 to 2023. The incidence of all lower limb amputations increased from 2019 to 2022 (23.9 vs. 25.7 %, p=0.01) with no significant changes in 2023. Moreover, the incidence of minor amputations increased from 16.9 % in 2019 to a maximum of 18.9 % (p<0.001) in 2022, and then to 18.4 % (p=0.02) in 2023. In the short-term perspective, inpatient cases significantly decreased in phases 1 and 3, with a relevant increase in the subsequent phases. However, the incidence of all amputations, especially minor ones, is constantly increasing.

**Conclusions:**

The COVID-19 pandemic has significantly indirectly impacted the management of CLTI patients, with a marked increase in the incidence of minor amputations, presumably due to the delayed presentation of patients with small necroses and due to limited access to healthcare.

## Introduction

The COVID-19 pandemic has had health, economic, and social effects on the population, which may cause short- and at least medium-term consequences. The WHO reported 5.42 million COVID-19-associated deaths in 2020–2021 but estimated 14.83 million deaths due to excess mortality [1]. The discrepancy between the numbers is explained by underestimating the number of deaths due to COVID-19 infection as well as the pandemic’s side effects. Different countries have adopted different strategies in the fight against SARS-CoV-2 infections, which in many cases limited the mobility of the population and their access to healthcare. The fear of infection and implementation of lockdown requirements might delay the presentation of patients in critical conditions, with postponed diagnosis and therapy initiation. Mazidimoradi et al. showed that general colorectal cancer screening decreased by 28–100 % in different countries after the outbreak of the COVID-19 pandemic [2]. Although the number of new diagnoses of colorectal carcinoma has decreased, the number of emergency presentations due to obstructions or perforations of the large intestine or presentations at an advanced stage of the disease has increased [3].

Critical limb-threatening ischaemia (CLTI) refers to acute decompensation of chronically reduced leg perfusion, defined by ischaemic rest pain and trophic lesions on the legs, and affects 11.08 % of patients with peripheral arterial disease (PAD) per year [4]. Despite modern therapies, amputation is necessary for 20 % of those affected. Additionally, mortality rate in patients with critical limb ischaemia is up to 25 % [4]. Treatment of the mostly frail CLTI patients includes complex management with vascular imaging, revascularisation of the lower limb to improve perfusion deficits and treat ischaemic sequelae such as rest pain and trophic lesion, and in case of amputation, fitting with a prosthesis or medical footwear followed by rehabilitation. Due to the high mortality rate, the high risk of limb loss, and the high treatment and rehabilitation costs with social implications after ablation, PAD, particularly at the stage of critical limb-threatening ischaemia, is becoming increasingly important and remains a central issue among vascular diseases. The care of patients with critical limb perfusion involves interdisciplinary teams and requires multimodal therapy, which can be susceptible to insufficient treatment when resources are limited. Considering the limited access of patients to healthcare in Germany during the COVID-19 pandemic, we hypothesise that the outbreak of SARS-CoV-2 significantly increased the amputation rate among patients with CLTI.

## Materials and methods

The evaluation is based on open access, anonymised data from the Institute for the Hospital Remuneration System (InEK) data browser (https://datenbrowser.inek.org) and data from the Robert-Koch-Institute (RKI=the government’s central scientific institution in the field of biomedicine, https://www.rki.de/DE). Special permission for data use and an ethics vote is not required for fully anonymised data. No patient-specific conclusions can be drawn from the data. According to § 21 KHEntgG (Hospital Fees Act), public data is freely available for scientific purposes. The clinical and epidemiological data are analysed at the federal level at weekly intervals to ensure comparability with the absolute numbers and 7-day incidences of COVID-19 patients according to the information provided by the RKI. According to the RKI, the course of the COVID-19 pandemic is divided into phases 1–8 and extended with pre- and post-pandemic periods (phases 0 and 9) for the current analysis (Table 1) [5]. The seasonal variation of the data is analysed by recording the running calendar weeks and determining the number of non-working weekdays in at least 50 % of the federal states.

**Table 1:** Definition of the phases of the COVID-19 pandemic according to the Robert Koch Institute [5] modified for the current analysis.

Phase	Description	Start–end (calendar week)
0	Pre-pandemic period	Until 09/2020
1	First COVID-19 wave	10/2020–20/2020
2	Summer plateau 2020	21/2020–39/2020
3	Second COVID-19 wave	40/2020–8/2021
4	Third COVID-19 wave (VOC alpha)	9/2021–23/2021
5	Summer plateau 2021	24/2021–30/2021
6	Fourth COVID-19 wave (VOC delta)	31/2021–51/2021
7	Fifth COVID-19 wave (VOC omikron BA.1/BA.2)	51/2021–21/2022
8	Sixth COVID-19 wave (VOC omikron BA.5)	22/2022–18/2023
9	Post-pandemic period	From 19/2023

Patients with CLTI are characterised by rest pain in the leg and/or ulceration or gangrene on the lower extremity and are defined respectively as I70.23, I70.24, I70.25 according to the International Classification of Diseases ICD-10-2019/-2023 for atherosclerosis of arteries of extremities pelvic-limb type. Cases with acute limb ischaemia (I74.0–5, I74.8) are excluded so that the impact of the COVID-19 pandemic on chronically ill patients can be specifically investigated. However, it remains uncertain whether these two conditions are consistently separated by diagnosis recording. Patients are defined according to the operation and procedure code OPS-2019/-2023 with the distinction between major amputations (5-864.0-9/a/x/y) and minor amputations (5-865.0-8/90/91/92/x/y) after a lower limb amputation. The general health status of patients is classified by the Patient Clinical Complexity Level (PCCL), which is calculated in a complex procedure from the values of the secondary diagnoses and recorded between 0 (no degree of complication or comorbidity) and 6 (most severe degree of complication or comorbidity).

### Statistical analysis

InEK and RKI data were linked in an Excel database (Microsoft Excel for Mac, version 16.63.1, Microsoft Corp., Seattle, WA, USA). The entire descriptive properties of the subgroups as well as the statistical analyses were performed with RStudio (2022.02.2, Build 485, 2019–2022, RStudio PBC, Prairie Trillium Release, 19.04.2022) for macOS after consultation with the Institute for Biostatistics and Mathematical Modeling at Goethe University Frankfurt am Main.

The statistical analysis includes a quantitative and qualitative analysis as well as the temporal dependencies between the number of COVID-19 cases and the treated patients with critical limb-threatening ischaemia. The analysed variables are examined with a Shapiro–Wilk test and graphically for the normal distribution. With p>0.05 and the assumption of a normal distribution, the variable comparison follows with the ANOVA test and post-hoc with a paired *t*-test. At p<0.05, the data are treated as non-normally distributed and analysed with nonparametric tests (Kruskal–Wallis test [KWT] and post-hoc a Dunn test). The correlation analysis was determined by a Pearson correlation coefficient (r) for normally distributed data and a Spearman correlation coefficient (ρ) for non-normally distributed variables. A correlation coefficient over 0.8 corresponds to a strong correlation, 0.6–0.8 to a moderately strong correlation and 0.3–0.6 to a fair correlation. The effects of the COVID-19 pandemic on the individual variables are determined using multiple linear regression and standardised data for comparability of the influencing strengths. The variables were examined for homoscedasticity, using a student Breusch–Pagan test. Applying the robust standard error according to the HC3 method corrected the heteroscedasticity of the data.

## Results

### Patient characteristics

The data analysis covers five calendar years, 2019–2023, with a total cohort of 476,168 patients (61.8 % male, 38.2 % female, <0.1 % diverse and unknown). Significant variations in the number of patients with CLTI can be observed over a year. Initially, there was a statistically insignificant decrease in the number of cases treated in 2020 (2019 vs. 2020, 96,482 vs. 92,006, relative decrease of 4.64 %, Dunn test p>0.05), followed by an increase with a significant difference between 2020 and 2023 (2020 vs. 2023, 92,006 vs. 99,002, relative increase of +7.07 %, Dunn test p=0.001). The proportion of men increased continuously from 2019 to 2023 (from a median of 60.9–62.1 %, ANOVA, p<0.001, r=0.36, p<0.001), while the proportion of women decreased continuously (from a median of 39–37.9 %, ANOVA, p<0.001, r=0.36, p<0.001) (Table 2).

**Table 2:** Epidemiological features of patients with critical limb-threatening ischaemia (CLTI) median (min; max).

Year	2019	2020	2021	2022	2023
CLTI cases per year	96,482	92,004	94,750	96,653	96,279
CLTI cases per week	1,879.5 (192; 2,363)	1,782 (358; 2,416)	1,864 (405; 2,163)	1,818.5 (297; 2,184)	1,910.5 (240; 2,258)
Male, %	60.9 (58.3; 67.2)	61.3 (58.5; 65.5)	61.4 (57.7; 64.2)	62.5 (59.9; 65.8)	62.1 (59.9; 65.4)
Female, %	39.0 (32.8; 41.7)	38.7 (34.5; 41.5)	38.7 (35.9; 42.3)	37.5 (34.3; 40.1)	37.9 (34.6; 40.0)

**Figure 1:**
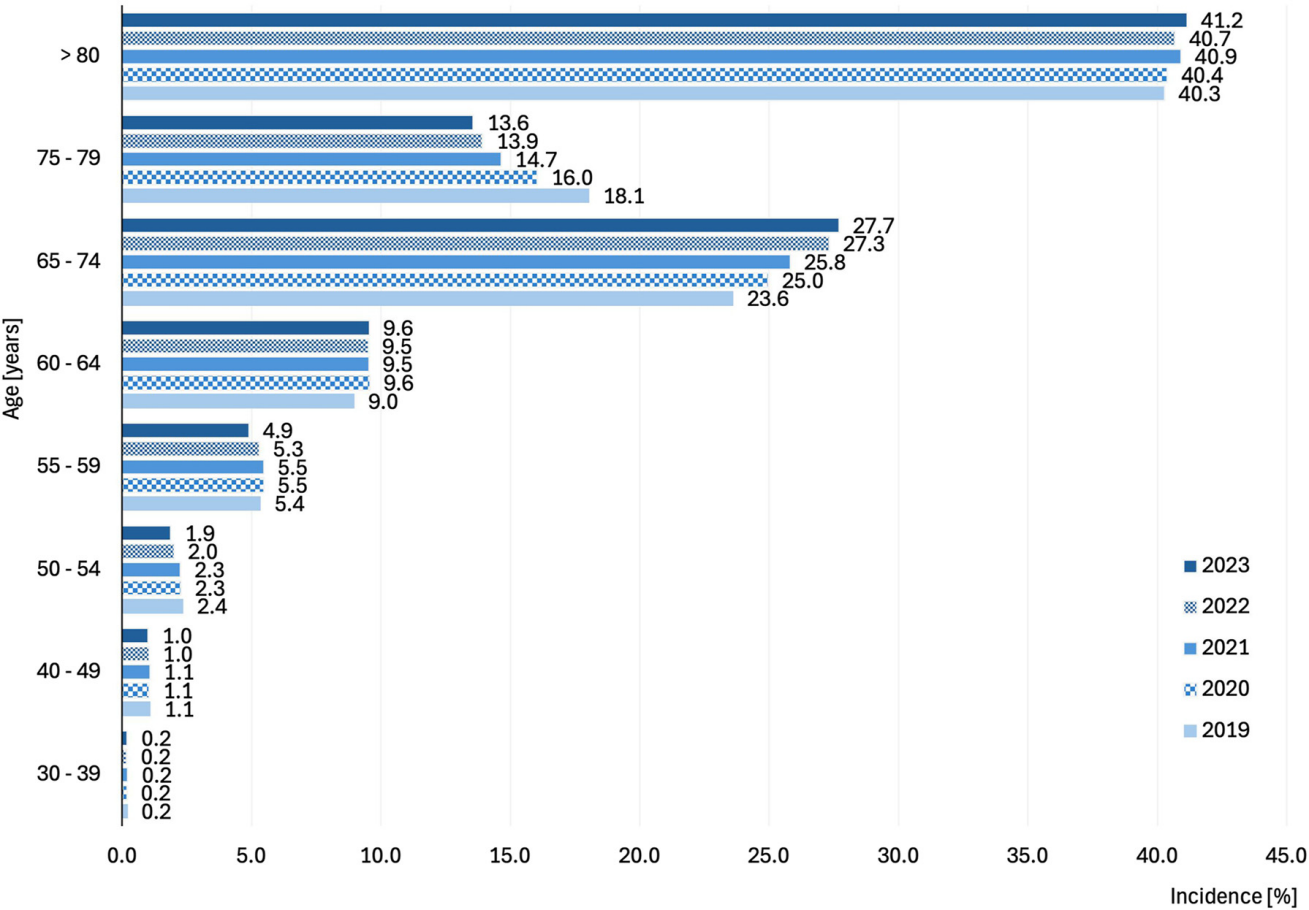
Age distribution of patients with critical limb-threatening ischaemia in the observed period 2019–2023.

CLTI is a disease of elderly patients. Patients aged over 80 years cumulatively account for 40.6 % of all CLTI patients from 2019 to 2023, followed by those aged 75–79 years (15.3 %), 65–74 (26.0 %), 60–64 (9.4 %), 55–59 (5.5 %) and 50–54 (2.1 %). Patients under the age of 50 make up only 1.3 % of all CLTI patients. Compared to the previous year (Figure 1), the proportion of patients aged 50–54 years continuously declines (from a median 2.4–1.9 %, KWT p<0.001, ρ= −0.45, p<0.001), 55–59 years (from median of 5.4–4.9 %, ANOVA p<0.001, r= −0.26, p=0.02), 75–79 years (from median 18.1–13.6 %, KWT p<0.0001, ρ= −0.82, p<0.001).

The increase in the proportion of patients with CLTI is observed in the group of patients aged 60–64 years (from a median 9.0–9.4 %, KWT p<0.001, ρ=0.19, p=0.002), 65–74 (from median 23.6–27.7 %, KWT p<0.001, ρ=0.19, p=0.002) and those aged over 80 years (from a median 40.3–41.2 %, KW test p=0.01, ρ=0.21, p<0.001).

**Figure 2:**
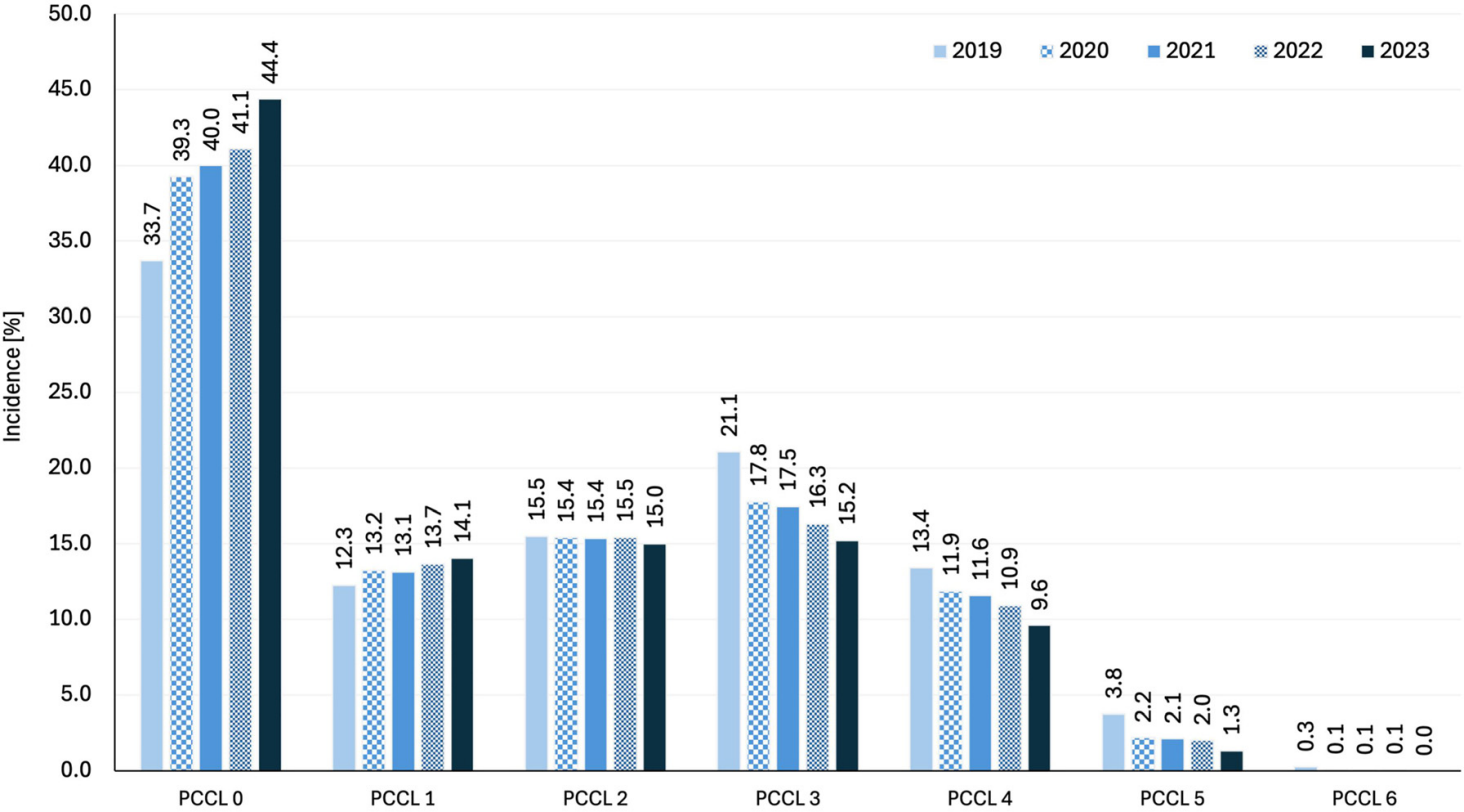
Classification of patients with chronic limb-threatening ischaemia depending on the patient clinical complexity level (PCCL).

Depending on the severity of complications and comorbidities, an average of 40.2 % of patients with CLTI were assigned to PCCL 0, 13.3 % to PCCL 1, 15.4 % to PCCL 2, 17.5 % to PCCL 3, 11.3 % to PCCL 4, 2.2 % to PCCL 5, and 0.1 % to PCCL 6 between 2019 and 2023 (Figure 2).

The proportion of patients with PCCL 0 increased significantly from 33.7 to 44.4 % (KWT p<0.001, ρ=0.77, p<0.001), and PCCL 1 from 12.3 to 14.1 % (ANOVA p<0.001, r=0.45, p<0.001) (Figure 2). From the PCCL 2 group onwards, the number of severely ill CLTI patients significantly decreased (ρ= −0.17, p=0.005) and a clear decrease from the PCCL 3 to 6 group onwards (ρ= −0.79, −0.66, −0.68, −0.55 with p<0.001 for all PCCL 3–6 groups).

### Characteristics of admission diagnoses

**Figure 3:**
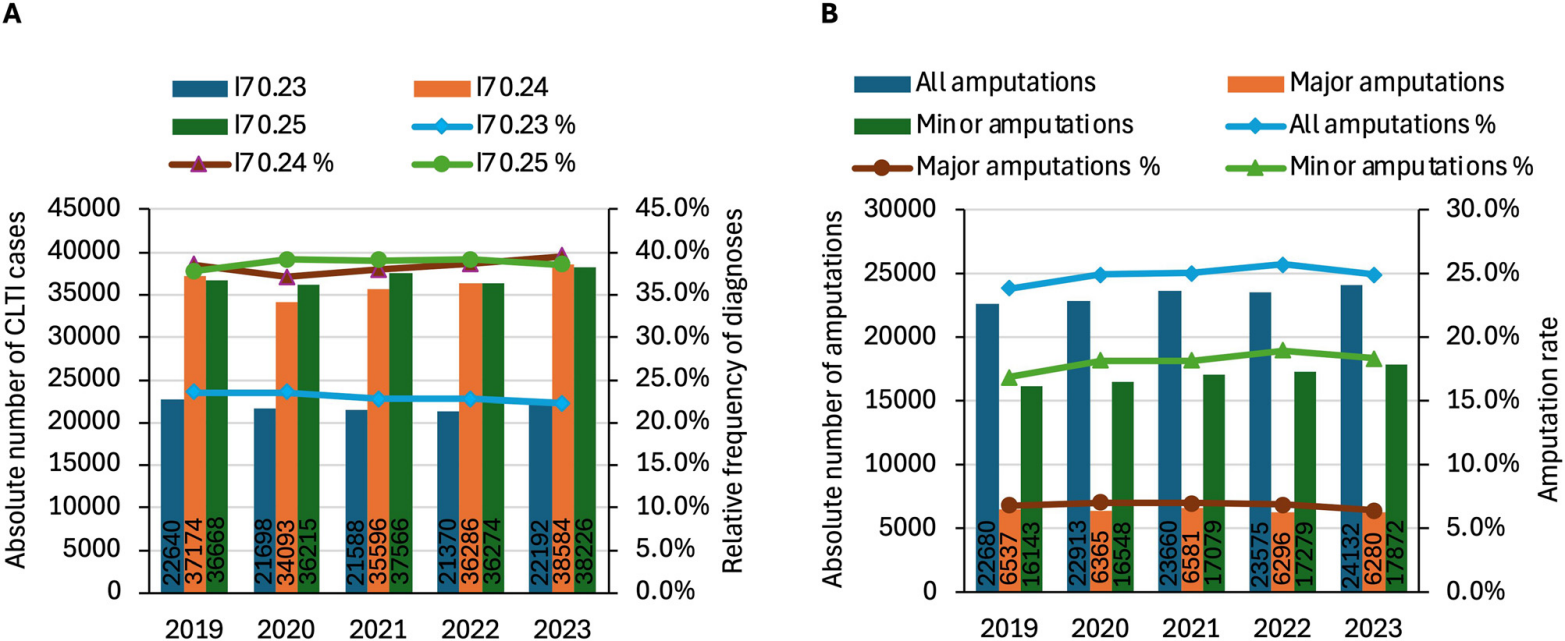
Annual distribution of chronic limb-threatening ischaemia (CLTI) diagnoses (atherosclerosis of arteries of extremities: pelvic-limb type with pain at rest (I70.23) with ulceration (I70.24), with gangrene (I70.25)) (A) and amputations (B).

Patients with CLTI were admitted primarily due to chronic ischeamic rest pain (23.0 %), with ulcerations (38.2 %) and gangrene (38.8 %) of the lower extremity (Figure 3A).

The total number of patients with rest pain (I70.23) and gangrene (I70.25) remained stable over the years (ρ<0.03, p>0.05). The number of patients with ulcerations initially decreased from 2019 to 2020 (relative decrease of −8.3 %, Dunn test p=0.01) and increased by 13.2 % from 2020 to 2023 (Dunn test p<0.001). However, due to the variable total number of all CLTI patients, the incidence of patients with rest pain decreased in the medium term (from 23.5 % in 2019 to 22.3 % in 2023, KWT p<0.001). The incidence of patients with ulcerations (I70.24) initially fell significantly from 2019 to 2020 (Dunn test p<0.001) and then rose from 2020 to 2023 above the initial value in 2019 (Dunn test p<0.001). The incidence of patients with gangrene (I70.25) initially increased significantly from 2019 to 2020 (Dunn test p=0.03) and remained the constant until 2023.

### Characteristics of lower limb amputations

Of 476,170 hospitalised patients with CLTI in the period 2019–2023, 24.6 % of cases required a lower limb amputation, of which 6.7 % were major and 17.8 % were minor amputations. The number of all amputations, major and minor, decreases gradually over each year with increasing calendar weeks (ρ= −0.74, −0.64, −0.71, p<0.001).

The total number of all amputations shows an increasing trend in the longer term (KWT p=0.07, ρ=0.16, p=0.008), which is due to the significant increase in the number of minor amputations (from 16,143 in 2019 to 17,872 in 2023, KWT p=0.002) by the constant number of major amputations (KWT p=0.4) (Figure 3B). On considering the fluctuations in the total number of patients treated with CLTI, the incidence of all lower limb amputations increased from a median of 23.9 % in 2019 to 25.7 % in 2022 (Dunn test p=0.01) with no significant changes in 2023. With a stable incidence of major amputations in the period under investigation, the significant increase in the incidence of minor amputations (KWT p<0.001) from 16.9 % in 2019 over a maximum of 18.9 % (Dunn test p<0.001) in 2022 to 18.4 % (Dunn test p=0.02) in 2023 is triggered by the increase in all amputations.

**Figure 4:**
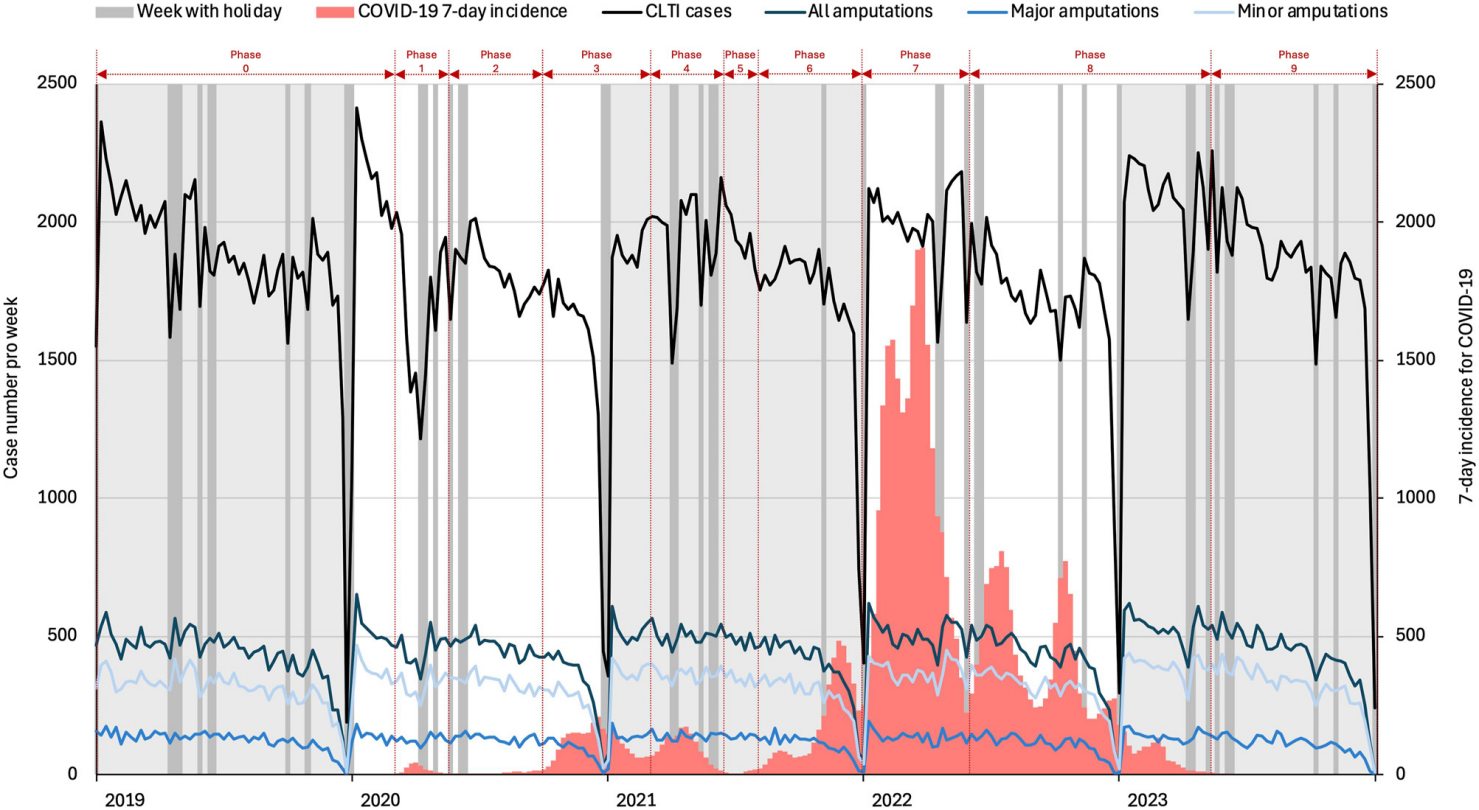
Seasonal and annual variations in cases of COVID-19, critical limb-threatening ischaemia (CLTI) and amputations.

The number of hospitalised cases with CLTI decreases gradually over each year (ρ=−0.64, p<0.001) and during the weeks with the holidays (ρ=−0.4, p<0.001). A significant decline in the number of cases is documented at the beginning and end of the year, corresponding to the Christmas holidays (Figure 4).

This Christmas break is offset by a sharp increase in inpatient CLTI cases as early as the second calendar week of each year. Other significant decreases in the number of cases can be observed during the Easter holidays, the week with Labour Day, Ascension Day and finally in the Whitsun holidays. Daylight saving time is characterised by stable weekly case numbers of patients with CLTI without sudden fluctuations. In autumn, two fall lows can be observed, caused by holidays such as the Day of German Unity and All Saints’ Day. In most cases, the decline in the number of cases is followed by an increase in the subsequent weeks.

In 2020, in addition to the typical fluctuations in case numbers around Christmas, a prolonged low in case numbers was noted from week 11 to week 25 due to the outbreak of the COVID-19 pandemic, with a smooth transition into the Easter and Whitsun vacations. Conversely, the autumn and winter-related fluctuations in case numbers were significantly flatter than in 2019. Unlike 2019 and 2020, 2021 began with lower case numbers and followed the pattern of the public and national holidays of 2019, with minor fluctuations in case numbers similar to autumn 2020. The distribution of cases treated in 2022 and 2023 corresponds to the seasonal fluctuations in the control group in 2019.

### Impact of the COVID-19 pandemic on the number of inpatient treatments and amputation rate

The investigation of the influence of the COVID-19 pandemic on the amputation rate in CLTI patients is complicated by the natural seasonal variation of inpatient cases throughout the year. The number of all amputations, as well as major and minor amputations, correlates with the number of treated patients with CLTI (ρ=0.78, 0.60, 0.77, p<0.001). The number of CLTI patients drops significantly in phases 1, 3, and 7 and increases significantly after the end of the COVID-19 pandemic (phase 9). Additionally, the number of CLTI patients depends on the holidays and decreases gradually over each year (Table 3).

**Table 3:**
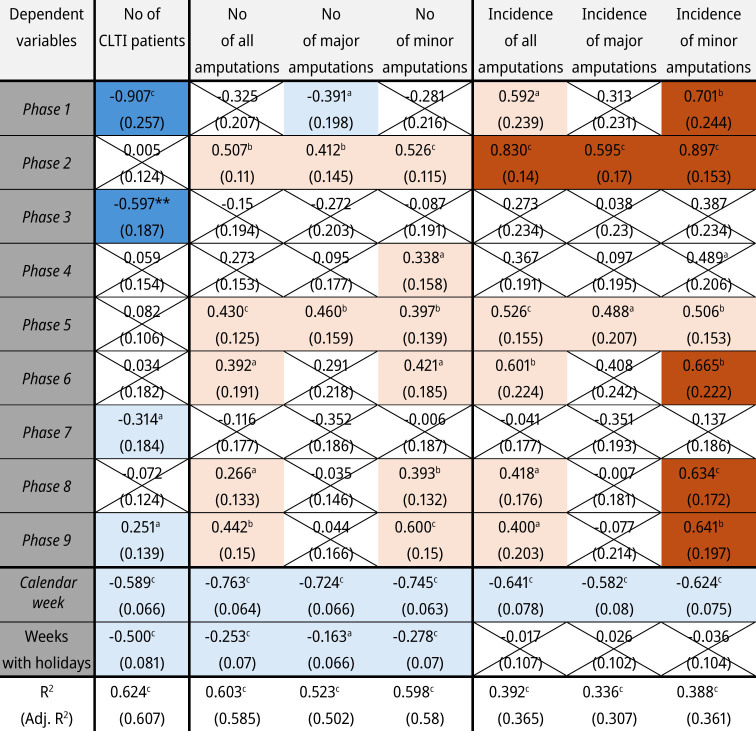
Multiple linear regression for the absolute number (No) of patients with critical limb-threatening ischaemia (CLTI), of amputations, and their incidences.

The light colours mark the significant effects of independent variables 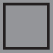, which influence the dependent variables 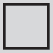 positively 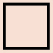 or negatively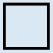. The darker colours mark additionally the significant effects of phases 1–9, which outweigh the dependent variables 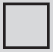 of prepandemic period (phase 0) as well as influence of calendar weeks and weeks with public holidays positively 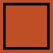 or negatively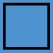. All results are presented after standardisation – results contain estimate, standard error in brackets, and significance with asterisks: ^a^p<0.05, ^b^p<0.01, ^c^p<0.001.

Both the absolute number of COVID-19 patients in the population and a 7-day COVID-19 incidence have no influence on the number of all amputations, as well as major and minor amputations. However, despite the low number of treated CLTI patients, the number of amputations during the first COVID-19 wave (phase 1) is not proportionately decreased, increasing the incidence of all amputations due to the rise in minor amputations. By the summer plateau of 2020 (phase 2), with stable numbers of inpatient treatments, an increase in both major and minor amputations is documented, further raising the incidence of amputation. In the second wave of the COVID-19 pandemic (phase 3), the inpatient treatment of CLTI patients was significantly reduced, but the absolute number and incidence of amputation remained at the same level. Throughout the COVID-19 pandemic, we observe stable (phase 7) or increasing (phases 4, 5, 6, 8, 9) numbers and incidences of amputations, especially for minor amputations, despite fluctuations in the number of inpatient treatments.

## Discussion

The influence of the COVID-19 pandemic on the health status of the population can be considered on multiple levels. The pathological influence of SARS-CoV-2 infection directly impacts structural and functional lung disease, deterioration and development of heart failure, intellectual performance, mental illness, thromboembolic diseases, and renal function. While the development of the aforementioned conditions can be directly linked to the viral disease, changes in the incidence of other diseases may be the consequence of limited access to healthcare and behavioural adaptation of the population, including the vascular patients. Yamamoto et al. reported a reduction in hospital admissions for cardiovascular diseases in 2020, with an incidence rate ratio of 0.88 for acute aortic dissection and 0.75 for ruptured aortic aneurysm compared with 2019 in the Tokyo Cardiovascular Care Unit Network [6]. UK AS Research Group presented a rapid reduction in aortic surgery during the first lockdown after the outbreak of the COVID-19 pandemic, with the near disappearance of elective surgery for abdominal aortic aneurysm, but still with sufficient management of emergency or urgent cases by constant aneurysm related mortality in the UK [7]. Bette et al. documented a −1.5 % decrease in inpatient treatments for unruptured aortic aneurysms in Germany from 2019 to 2021, with the maximum decrease in the first COVID-19 wave of −25.5 %, but without an increase in cases as a rebound in 2021, suggesting an accumulation of untreated abdominal aortic aneurysms in the population [8]. The postponement of carotid endarterectomy was attributed to administrative decisions in the USA pertaining to COVID-19 protective measures in 72 % of cases, with a median delay of 71 days in 69 % of asymptomatic and 31 % of symptomatic patients. Despite the stability of the neurovascular disease, 5 % of patients died while awaiting surgery [9]. The immediate impact of the pandemic on standard care of CLT I patient was shown in the Italian population by the reduction in vascular operations by 0.8–40 % in the first and second wave of COVID-19 [10]. However, in all the vascular diseases described above, the long-term reports on the effects of the COVID-19 pandemic are missing. Our analysis captures the largest cohort of 476,168 patients treated in the German healthcare system, with the longest observation and documentation of the immediate or medium-term effects in the context of the COVID-19 pandemic. Noory et al. attempted to map the pandemic effects in CLTI patients, but their recorded experiences concerned a single German centre cohort and the numbers when the pandemic was ongoing, underestimating delayed effects and global cross-regional processes [11].

A significant increase in CLTI incidence in males and a reduction in the proportion of females during the observation period from 2019 to 2023 is striking and documented for the first time. However, Song et al. presented higher CVD-related mortality in 2019–2021 by males than females [12], which is interpreted by a hormonal, genetic and behavioral disadvantage of men [13]. Because males are already more susceptible to cardiovascular disease in advance, limited access to healthcare combined with the negative biological impact of COVID-19 infections on vascular disease may accelerate the progression or reduce the detection of PAD in the early stages.

The COVID-19 pandemic was associated with a change in the age distribution of CLTI patients. CLTI continues to be a disease of elderly patients, predominantly those over 80 years of age. Furthermore, we observe a reduced incidence of patients younger than 60 years of age, with an increase in inpatient admissions among patients aged 60–74 and over 80 years. Surprisingly, the incidence of inpatient CLTI cases has fallen in the 75–79 age group. However, we do not find any reports that performed a subgroup analysis in such small age intervals that we did. As all other groups of patients over 60 years of age in our cohort followed the typical reported course of cardiovascular diseases, we observe probably a random statistical effect in the 75–79 age group [12].

The COVID-19 pandemic negatively impacted not only major adverse limb events (MALE) but also major adverse cardiovascular events among Italian patients [14]. The oncological Dutch patients were operated during the COVID-19 pandemic at the more advanced stage of cancer and by higher rate of comorbidities than in the presurge [15]. Our analysis, on the other hand, documents a decrease in the number of critically ill CLTI patients (PCCL 3–6) and the dominance of patients with few comorbidities. If the lower proportion of severe ill patients with CLTI could be explained by the effect of preclinical triage under reduced care capacities in 2020 and 2021, this argument no longer explains the still reported downward trend from 2022 onwards.

Our analysis not only presents the pandemic’s influences on the national care of CLTI patients but also reveals a consistent distribution of patients throughout each year, with notable seasonal fluctuations, particularly around the holidays. The nationwide fluctuation in the number of treated CLTI patients in German society is supported by single-centre observations [16] and the North American registry [17], although the available data primarily pertain to the early phases of the COVID-19 pandemic. However, by extending the observation period, we were able to differentiate the changes of amputation statistics in the individual COVID-19 phases from the usual effects over the course of the calendar year using multiple linear regression. Since the 7-day COVID-19 incidence and the number of COVID-19 cases show no correlation with the number of hospitalised CLTI patients or amputation rates, the focus should perhaps shift from the pandemic itself to factors such as resource redistribution, limited access to healthcare, and changes in patient behaviour as the primary causes of the medium-term rise in minor amputation incidences. Conversely, the stable number of major amputations, despite the pandemic and its related effects, may suggest that the national healthcare system has remained sufficiently robust, providing reliable care for critically ill patients. The impact on the care of CLTI patients was most evident in periods of increased mobility restrictions and protective measures during the first and second lockdowns, corresponding to the first and second COVID-19 waves (phases 1 and 3), which is reflected in the lower total number of treatments. The effects of delayed patient care are evident in the increased incidence of amputations, particularly during the intervals between COVID-19 waves or following the lifting of lockdowns, when wider access to outpatient and inpatient care resumed, as seen in phases 4, 5, 6, 8, and 9.

It remains important to define the factors that increased the incidence of amputations in the first waves of the COVID-19 pandemic. Zayed et al. demonstrated in a single-centre analysis a delayed admission of patients with CLTI and diabetic foot in more advanced stages [18]. This observation is confirmed in our analysis, as the incidence of patients with rest pain has decreased, but the incidence of patients with ulcerations and gangrene has increased. Rando et al. pointed out that these patients were admitted not only in a more advanced stage of the disease but also as emergencies via the emergency room, instead of electively via a polyclinic [14]. These aspects could explain the higher amputation rates at the lower extremity in the surge compared with the presurge months in the Italian cohort [14].

Structural adjustments prioritizing the care of COVID-19 patients led to a reallocation of resources from other areas of the healthcare system, resulting in insufficient or delayed care for non-COVID-19 patients. The North American registry reports that vascular procedural volumes for CLTI patients were 15.9 % [17]. Although patient care during the COVID-19 pandemic was delivered more quickly, the outcomes were less favorable, with lower primary patency rates, reduced freedom from MALE, and poorer amputation-free survival and limb salvage compared to pre-pandemic levels [18].

The German healthcare system has demonstrated efficacy in the treatment of critical limb-threatening ischaemia under constrained resources. However, the preliminary stages of PAD decompensation remain underestimated, which may contribute to the rising incidence of minor amputation due to delayed patient care. It remains unclear whether this phenomenon is the result of behavioral adaptations by patients or a reduction in healthcare services during the pandemic. Raising patient awareness of the precursors of critical illnesses through health education and early reactivation of general health checks with preventive examinations of risk groups are the first measures to detect critical leg perfusion and increase the chance of leg preservation.

## Conclusions

The COVID-19 pandemic altered the demographics of CLTI patients, leading to an increase in the number of men and older patients being treated, while fewer patients with multiple comorbidities were hospitalized. COVID-19 itself had no direct impact on amputation rates in Germany. However, the incidence of minor amputations rose, particularly after periods of strict lockdowns and in long-term follow-up, suggesting that patients’ behavioural adaptations in response to limited healthcare access played a significant role.

## Limitations

Disease stage was determined using DRG information and is related to the Fontaine classification of peripheral arterial disease, which may result in unrecognised overlap with acute limb ischaemia, diabetic complications or non-atherosclerotic causes of trophic lesions in the legs. The data is fully anonymized and pooled, which limits the potential for subgroup analysis. The database does not allow further tracking of individual patients to analyse mortality and morbidity in the combination of CLTI and COVID-19 disease.
